# An extensively hydrolysed protein-based extruded diet in the treatment of dogs with chronic enteropathy and at least one previous diet-trial failure: a pilot uncontrolled open-label study

**DOI:** 10.1186/s12917-025-04528-y

**Published:** 2025-02-15

**Authors:** Valérie Freiche, Olivier Dossin, Amélie Leclerc, Isabelle Mougeot, Jeremy Laxalde, Olivier Roy, Vincent Biourge, Alexander J. German

**Affiliations:** 1 Unité de médecine interne, Ecole Nationale Vétérinaire d’Alfort, CHUVA, Maisons-Alfort, France; 2 Laboratory of Cellular and Molecular Mechanisms of Hematological Disorders and Therapeutical Implications, INSERM, Imagine Institute, U1163, University of Paris, Paris, France; 3 ADVETIA, Small Animal Internal Medicine, Velizy-Villacoublay, 78140 France; 4 Centre Vétérinaire Daubigny, Québec, Canada; 5 Symrise, Elven, France; 6 Royal Canin Research & Development, Aimargues, France; 7 Cebiphar, Fondettes, 37230 France; 8 Institute of Life Course and Medical Sciences, University of Liverpool, Liverpool, UK; 9 Clinique Vétérinaire Alliance, Bordeaux, France; 10 National Veterinary School of Toulouse, Toulouse, France

**Keywords:** Food-responsive enteropathy, Inflammatory bowel disease, Nutrition, Hypoallergenic, Oligopeptide, Amino acids, Protein hydrolysate, Chronic diarrhoea

## Abstract

**Background:**

Canine chronic enteropathies (CE) are a group of disorders defined by persistent or recurrent clinical signs of gastrointestinal disease without a primary neoplastic, metabolic, parasitic, or other infectious cause. In this prospective, multicentre, uncontrolled, open-label study, a commercial dry diet with a protein source of extensively hydrolysed poultry feather was assessed in the management of dogs with CE that had not responded to previous dietary and antibacterial therapies. Dogs with moderate or marked protein-losing enteropathy were excluded. After screening, dogs entered stage 1 and started the test diet. Gastrointestinal endoscopy was performed, and only dogs with histopathological evidence of small intestinal inflammation confirming CE could continue to stage 2 of the trial. The test diet was fed for 10 weeks throughout stages 1 and 2, and the primary outcome measure was clinical success defined as a reduction in canine inflammatory bowel disease activity index (CIBDAI) of ≥ 75%. Secondary outcomes included body condition score (BCS, scale 1–9) and faecal consistency score (scale 1–5). Results (median [range]) for dogs with confirmed CE that participated in both study stages are reported.

**Results:**

A total of 15 dogs commenced stage 1, and 13 of these progressed to stage 2 (age 4.2 [1.1–7.1] years; BCS 3 (2–4); previous diet therapies 2 [1–3]) of which two were withdrawn at week 5 for protocol deviations. CIBDAI scores decreased from 9 (7–16; *n* = 13) at baseline to 2 (1–11; *n* = 13) at week 2 (*P* < 0.001), 2 (0–6; *n* = 13) at week 5 (*P* < 0.001), and 1 (0–3; *n* = 11) at week 10 (*P* < 0.001). Treatment success was achieved by 8/13 dogs at week 5 and 10/11 dogs at week 10. Faecal score (*n* = 11) and BCS (*n* = 11) improved between baseline (1 [1–3] and 3 [3–4], respectively; *P* < 0.001) and week 10 (4 [3–5] and 4 [3–5], respectively; *P* < 0.001).

**Conclusions:**

Dogs with CE that had failed to respond to previous dietary and antibacterial therapy showed clinical improvement within 10 weeks when fed a dry extruded diet with a single protein source hydrolysed to amino acids and oligopeptides, without concurrent immunosuppressant treatment.

**Supplementary Information:**

The online version contains supplementary material available at 10.1186/s12917-025-04528-y.

## Introduction

Canine chronic enteropathies (CE) are a group of disorders that are defined by persistent or recurrent clinical signs of gastrointestinal disease without a primary neoplastic, metabolic, parasitic or other infectious cause [1]. Various clinical signs reported include vomiting, diarrhoea, tenesmus, mucoid stools, haematochezia, weight loss, abdominal pain and anorexia for a period of more than 3 weeks [1–4]. In a recent retrospective study, almost 65% of dogs presenting with chronic diarrhoea were ultimately diagnosed with CE [4]. Although the aetiology of CE is unknown, it is thought to be the result of complex interactions between host genetics, the immune system, and gut microbiota [3, 5–8]. Cases of CE are usually sub-categorised according to therapeutic response: food-responsive enteropathy (FRE), antibiotic-responsive enteropathy (ARE), immunosuppressant-responsive enteropathy (IRE), and non-responsive enteropathy (NRE) [1, 7, 9]. In addition, both IRE and FRE can be associated with protein-losing enteropathy (IRE-PLE and FRE-PLE, respectively) [10]. Food-responsive enteropathy is the predominant type of CE [1, 11] and is more likely to affect younger dogs and be associated with less severe clinical signs than IRE [12, 13]. Dietary trials, using either a single-source protein or hydrolysed protein food, are required both for diagnosis and as first-line therapy [3, 4, 9, 14, 15], and most dogs respond without the need for immunomodulatory therapy [12, 16]. Traditionally, when the response to dietary trials is poor, antibacterial trials are considered, and those dogs responding neither to diet nor antibacterials are classified as having suspected IRE. More recently, it has been proposed that empirical antibacterial treatment should be reserved for cases in which all other conditions have been excluded and empirical treatments exhausted [17].

Immune dysregulation in FRE manifests as a loss of tolerance to dietary protein [5]. An IgE-mediated hypersensitivity type I reaction to a protein-derived food allergen, causing mast cells to degranulate and release heparin and histamine, is a potential but unproven component of the pathogenesis [18–20]. Many other innate or adaptive immunological mechanisms potentially play different roles in CE in combination with increased permeability of the intestinal epithelium, gut dysbiosis, and a genetic predisposition [8, 10, 21]. Immunological findings in dogs with CE include, for example, increased numbers of B lymphocytes in the bloodstream and intestinal mucosa [22], reduced IgA concentration in the intestinal mucosa [5, 23], a decrease in duodenal T-regulatory cells [24], and an increase in intestinal T cell receptor γδ-positive intraepithelial lymphocytes [25]. The size and structure of dietary protein influences its ability to induce an antibody-mediated hypersensitivity response [19]. In humans, CD4 T helper cells recognise antigens of 13–17 amino acids length presented by human leucocyte antigen (HLA) class II [26], the average B-cell epitope is about 15 amino acids [27], and mast cell activation requires an antigen with at least two IgE binding sites to enable Fcε receptor I cross-linking [28]. Whatever the exact immunological mechanisms in FRE, therefore, there is a biological rationale for hydrolysis of dietary proteins to create oligopeptides smaller than those likely to be immunogenic [18]. In addition, remission of CE induced by a hydrolysed protein diet is associated with improved composition of the microbiota in the gut, although it is not clear whether this is a cause, effect or a combination of both [8].

Several therapeutic diets with a hydrolysed protein source are now commercially available to assist with the management of CE. These diets differ in the nature and extent of the protein hydrolysis that has taken place. The first generation of hydrolysed proteins were produced through an enzymatic process, resulting in oligopeptides with mean molecular weights of 6–12 Kd, and such diets can be effective in managing canine CE [18, 29, 30]. However, smaller peptides than this are even less likely to be recognised as antigenic epitopes, with free amino acids and peptides less than 1 Kd not being recognised by the immune system [18, 31]. An elemental diet based on free amino acids has been tested in dogs with uncontrolled CE; 16/23 dogs were in clinical remission after 8 weeks of feeding [32].

The current study trials a therapeutic diet developed with a second-generation hydrolysate comprising poultry feather (keratin) protein extensively hydrolysed with HCl to form free amino acids (88%) and oligopeptides of < 1 Kd, i.e. 6–8 amino acids in length [33]. This oligopeptide and amino acid mix is not recognised by poultry-specific IgE [34], and its lack of “allergenicity” has been demonstrated by the absence of adverse reactions and pruritus when fed to dogs known to be sensitised to chicken protein [35]. Such a diet might well be of benefit in managing cases of CE, including those that have not responded to other types of food such as first-generation hydrolysed protein diets. Therefore, the primary aim of this study was to investigate the efficacy of a second-generation hydrolysed protein diet in management of canine CE that had previously failed to respond to other adequately-designed dietary and antibacterial trials.

## Results

### Study dogs and baseline variables

**Fig. 1:**
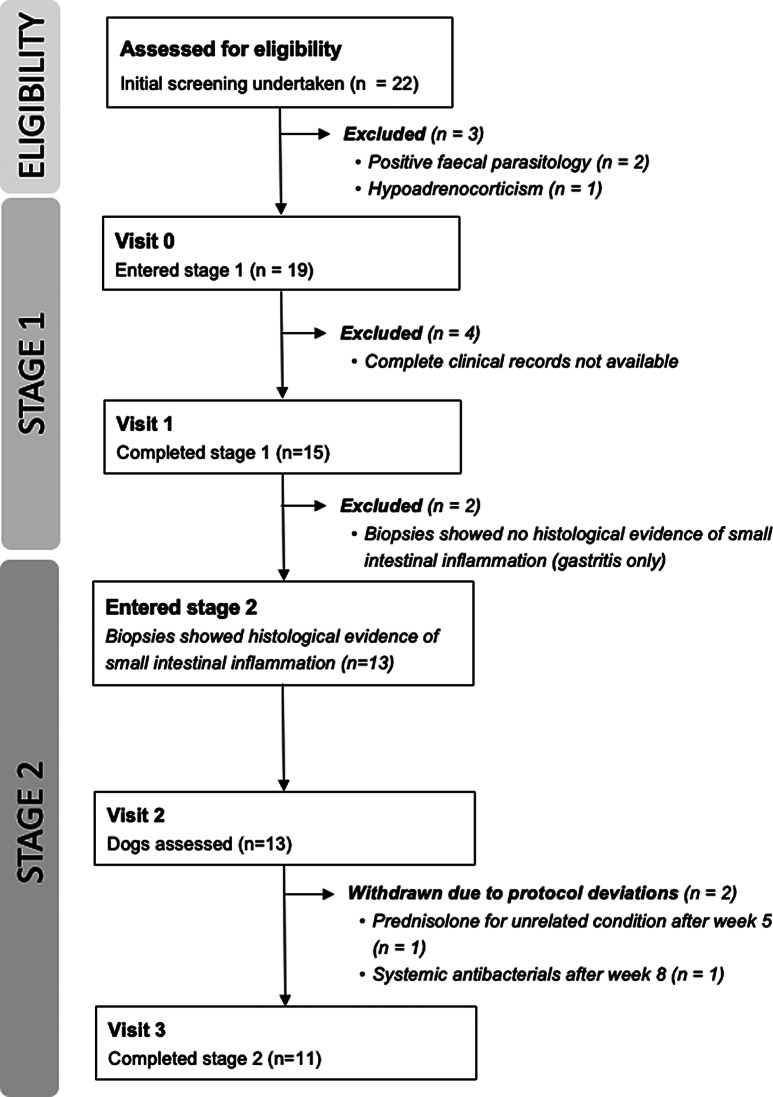
Study flow and subject disposition. Visit 0 took place no more than 7 days after the initial screening visit. Visits 1, 2 and 3 were scheduled for approximately 14 days, 35 days and 70 days after visit 0

Details of enrolment and study participation are given in Fig. 1. A total of 22 dogs were initially screened for eligibility; three were excluded, of which two had positive faecal parasitology results, and one was diagnosed with hypoadrenocorticism. The remaining 19 dogs of various breeds, ages and sexes were enrolled into stage 1 and commenced the study diet (visit 0), although four were subsequently excluded because complete clinical records were not available, meaning that 15 dogs completed stage (1) Two dogs completing stage 1 only had histological evidence of gastric inflammation and were therefore excluded from stage 2 of the study. Histological examination confirmed CE in the remaining 13 dogs and they progressed to stage (2) Only data for these 13 confirmed cases of CE are presented, although details of all dogs completing stage 1 or stage 1 and 2 are provided in Additional file 1. Baseline variables are presented in Table 1.

**Table 1:** Baseline characteristics and clinical history

Baseline variable	Dogs with confirmed CE
Number of dogs	13
French study sites	12
Canadian study site	1
Sex	
Male (intact / neutered)	7 / 1
Female (intact / neutered)	2 / 3
Age (years)	4.2 (1.1–7.1)
Breed	Bernese Mountain Dog, Border Terrier, Bichon Frise, Cane Corso, Coton de Tulear, Fox Terrier, Labrador Retriever, Mixed Breed (2), Pinscher, Shar Pei, West Highland White Terrier, Yorkshire Terrier
Body weight (kg)	9.8 (2.5–43.0)
Body condition score (/9)	3 (2–4)
Duration of clinical signs (months)	10.5 (1.3–55.7)
At least one previous diet trial ^a^	13
Diet type	
Highly digestible	13
Single-source protein ^b^	5
Hydrolysed protein	3
At least one prior antibacterial treatment ^c^	13
Antibacterial used	
Amoxicillin	1
Amoxicillin-clavulanate	1
Enrofloxacin	1
Metronidazole	11
Metronidazole & spiramycin	1
Phthalylsulfathiazole	1
Sulfaguanidine	2
Sulfaguanidine & framycetin	1
Sulfasalazine ^d^	1

Continuous data are reported as median (range). Categorical data are reported as number of dogs. ^a^ Dogs may have completed more than one trial in the same category. ^b^ A single-source protein diet was defined as a diet with whole animal protein from a single species (selected on the basis of that the dog had not previously been exposed to this protein), combined with carbohydrate from a single source. ^c^ Numbers represent dogs that received at least one course of the particular antibacterial, recognising that some dogs received more than one course of the same antibacterial. ^d^ Sulfasalazine is listed here as an antibacterial agent, although this drug and its metabolites also have immunosuppressive and anti-inflammatory activity

#### Clinical signs at baseline

All dogs (*n* = 13) had clinical signs of CE and the median duration of these at visit 0 was 11 (1–56) months. The most frequent clinical signs were diarrhoea (13/13), permanent or recurrent dysorexia (12/13), weight loss (11/13) and vomiting (10/13). Other clinical signs included borborygmus (7/13) and melaena (5/13). Four of the 13 dogs presented with haematochezia in addition to signs of small intestinal disease.

#### Previous dietary and antibacterial therapy

All dogs (*n* = 13) had previously received and failed dietary trials lasting at least 14 days, with the median being 2 (range 1–3) trials (Table 1). Seven dogs had received at least one single-source protein or hydrolysed protein diet in these trials (Additional file 1). All had received and failed one or more antibacterial trial (median 1, range1–4) lasting at least 14 days (Table 1). Details of the drugs used for these trials is given in Additional file 1.

#### Histological analysis of gastrointestinal tract biopsies

Endoscopy was performed a median of 7 (1–16) days after the initial examination. During the procedure, a median of 16 (11–34) biopsies were taken from different regions of the gastrointestinal tract. Biopsies were not obtainable for the duodenum in one dog and the ileum in two dogs. Histological results for all dogs with inflammatory changes in the small intestine are presented in Table 2.

**Table 2:** Gastrointestinal histological results from biopsies taken at visit 0 in dogs with confirmed chronic enteropathy

Histological lesions	Stomach	Duodenum	Ileum	Colon
Number of dogs with available specimens	13	12	11	13
No lesions present	1 (8)	1 (8)	1 (9)	2 (15)
Severity of inflammatory infiltrate ^a^				
Mild	11 (85)	6 (50)	3 (27)	5 (39)
Moderate	1 (8)	2 (17)	5 (45)	4 (31)
Severe	0 (0)	3 (25)	2 (9)	2 (15)
Fibrosis ^b^	4 (31)	0 (0)	1 (9)	
Helicobacter-like organisms	7 (54)	–	–	–
Villous atrophy	–	2 (17)	3 (27)	–
Signs of lymphangiectasia ^c^	–	2 (17)	1(9)	–

Results are reported as the number of dogs whose specimen had the reported finding, with the percentage in brackets (based on the total number of dogs with available specimens for that region). ^a^ Severity of inflammatory infiltrate was scored according to guidelines from the World Small Animal Veterinary Association (WSAVA) Gastrointestinal Standardization Group [60]. ^b^ All sides stained with haematoxylin and eosin, and reviewed by pathologists according to WSAVA criteria [60]; no staining specific for fibrosis (e.g., Masson’s trichrome) was used. ^c^ All cases of lacteal dilation were described as having mild lymphangiectasia

### Concurrent therapy, protocol deviations and study withdrawals

Of the 13 dogs that entered stage 2, two were subsequently withdrawn after visit 2; one dog was withdrawn because oral prednisolone therapy was required for an unrelated neurological condition, and the other dog developed superficial pyoderma and required cefalexin (Rilexine^®^ 300, Virbac Animal Health, 15 mg/kg twice daily, per os). Besides this, four dogs, all of which completed both stages, received diosmectite treatment; this treatment was administered between visit 0 and visit 1 in one dog, between visit 1 and 2 in two dogs and between visit 1 and 3 in one further dog. Parenteral cobalamin was administered to three dogs (at 50 µg/kg subcutaneously once per week for 6 weeks; Vitamin B12, Delagrange 1000 µg/mL), of which two completed all study visits and the remaining dog was withdrawn after visit 2 on account of developing superficial pyoderma as described above.

Other than diosmectite and cobalamin, various concomitant treatments were administered at the investigators’ discretion (Additional file 1). In some cases, existing medications were continued, including maropitant (Cerenia^®^, Zoetis; 1dog), omeprazole (Gastroguard^®^, Boehringer-Ingelheim: one dog), prifinium bromure (Prifinial^®^, Vetoquinol; one dog) and ursodiol (generic, one dog). In other cases, treatment with oral drugs was commenced, including activated charcoal (Carbolevure^®^, Pierre Fabre; one dog), milbemycin-praziquantel (Milbemax^®^, Elanco Animal Health, one dog), prifinium bromure (Prifinial^®^, Vetoquinol; one dog), sucralfate (Ulcar^®^, Sanofi Aventis; one dog) and tramadol (Tralieve^®^, Dechra; one dog). There was also some use of topical therapies, including chlorhexidine (Biseptine^®^, Bayer; one dog), imidacloprid-permethrin (Advantix^®^, Elanco Animal Health, one dog) and spinosad (Comfortis^®^, Elanco Animal Health, one dog).

Occasional minor dietary protocol deviations in five dogs were reported at visit 1, comprising consumption of cooked carrots (*n* = 1), cooked courgette (*n* = 1), fruits and flowers in the garden (*n* = 1), a piece of chicken (single occasion, *n* = 1) and increased rations of the study diet (*n* = 1). At visit 2, owners of six dogs reported consumption of crisp bread (*n* = 1), unspecified material from outside (*n* = 1), carrots (*n* = 2), mushrooms (*n* = 1), and the meal of another dog (single occasion *n* = 1). Dietary deviations for seven dogs reported at visit 3 were the feeding of crispbread (*n* = 1), carrots (*n* = 3), courgette (*n* = 1), rice (*n* = 1), and increased rations of the study diet ration (*n* = 1). As assessed by the owners, most palatability scores for the diet were good (i.e., ≥ 4; Additional file 1). Poor palatability (score 2) was only reported more than once for two stage 2 dogs. One stage 2 dog was reluctant to eat the diet (score 1) between weeks 2 and 4 but had no observed dietary protocol deviations and palatability improved subsequently, with scores of 3 or 5 for the remaining time points.

### CIBDAI

**Fig. 2:**
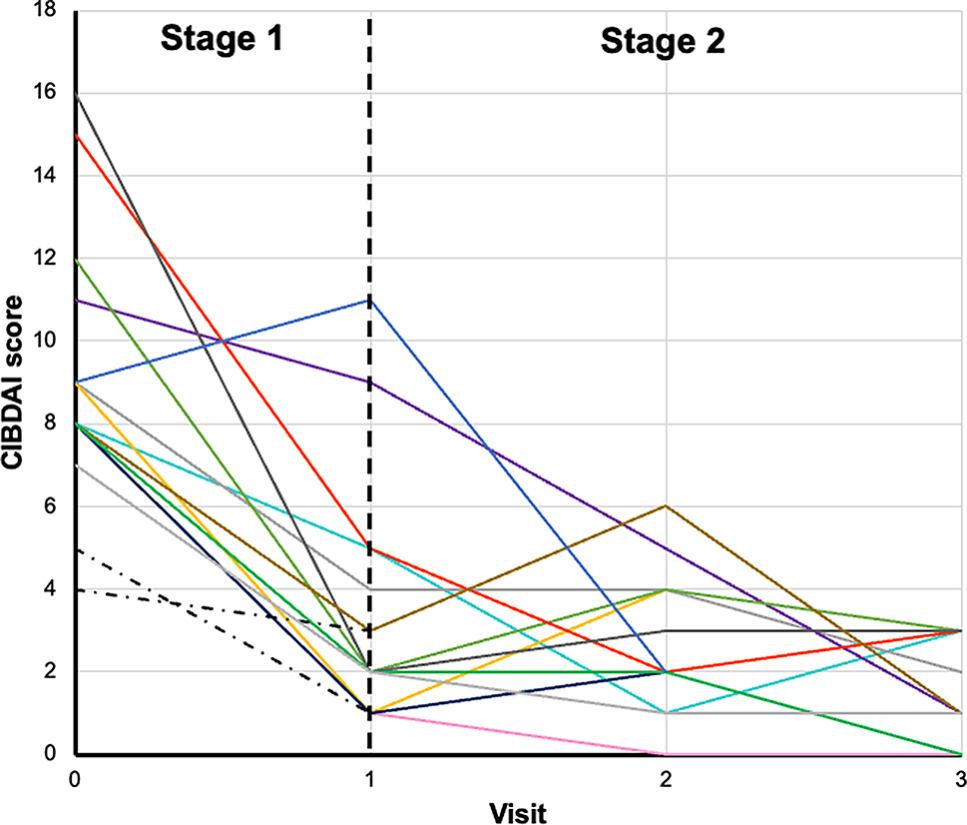
Chronic inflammatory bowel disease activity index (CIBDAI) scores over time. CIBDAI scores were assessed at visit 0 and visits 1, 2 and 3, occurring after approximately and 2, 5 and 10 weeks of feeding study diet, respectively. Each line represents an individual dog (*n* = 13)

Table 3 summarises the severity of clinical signs in study dogs according to CIBDAI score. During stage 1, median CIBDAI score decreased from 9 (range 7–16) at visit 0 to 2 (1–11) at visit (1) By visit 2 in stage 2, 8/13 (62%) dogs had achieved a ≥ 75% reduction in CIBDAI score from visit 0 and the median CIBDAI score was 2 (0–6). At study completion, 10/11 (91%) dogs had a ≥ 75% reduction in CIBDAI score and all had a CIBDAI score corresponding to ‘clinically insignificant disease’ (median 1 [0–3]). When CIBDAI scores were analysed across all visits (*n* = 13 dogs), a significant effect of visit was evident (*P* < 0.0001), with post-hoc analysis suggesting that scores at visit 0 were greater than those at each subsequent visit (*P* < 0.0001 for each comparison). The trajectory of changes in CIBDAI score for all dogs are illustrated in Fig. 2. In one of the two dogs withdrawn prematurely after visit 2, there was a transient increase in CIBDAI score between visits 0 and 1 (9 vs. 11), before decreasing to a score of 2 at visit 2. In the other dog, CIDBAI score was 9, 1 and 4 at visits 0, 1 and 2, respectively, suggesting mild disease at the time of exclusion for antibacterial treatment of pyoderma. This dog had also had a single dose of diosmectite 7 days after visit 0. In two of the other dogs treated with diosmectite, there were decreases in CIBDAI scores of a similar magnitude to other responders: CIBDAI scores at visits 0, 1, 2 and 3 were 8, 1, 2 and 0, respectively for the dog treated with diosmectite for 15 days (and thereafter as necessary), and 9, 4, 4 and 2, respectively for the dog that received diosmectite for 56 days. The remaining diosmectite-treated dog was treated for 22 days and responded more slowly with CIBDAI scores of 11, 9, 4 and 1, at visits 0, 1, 2 and 3, respectively.

**Table 3:** Canine inflammatory bowel Disease Activity Index score over time in dogs with confirmed chronic enteropathy

CIBDAI		Visit 0^a^ (*n* = 13)	Visit 1 (*n* = 13)	Visit 2 (*n* = 13)	Visit 3 (*n* = 11)
Score	Category	Number (%) of dogs			
0–3	Clinically insignificant	0 (0)	8 (62)	8 (62)	11 (100)
4–5	Mild	0 (0)	3 (23)	4 (31)	0 (0)
6–8	Moderate	5 (38)	0 (0)	1 (8)	0 (0)
≥ 9	Severe	8 (62)	2 (15)	0 (0)	0 (0)

Overall there was a significant effect of visit on CIBDAI score (*P* < 0.0001). ^a^Scores at visit 0 were higher than those at visits 1, 2 and 3 (*P* < 0.0001 for each comparison)

CIBDAI, Canine Inflammatory Bowel Disease Activity Index

### Body weight, body condition score, and appetite

**Fig. 3:**
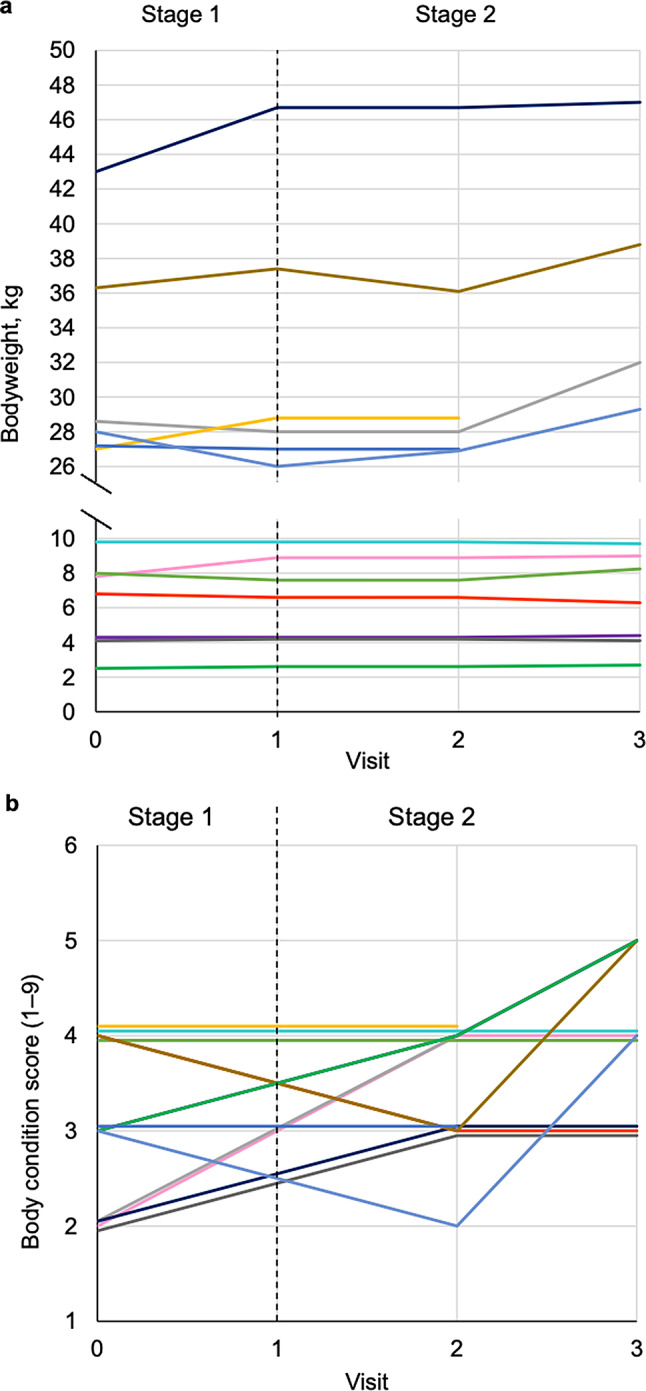
Changes in bodyweight (**a**) and body condition scores (**b**) in the 13 dogs completing the study. Dogs were weighed at all study visits and body condition score was assessed with a 9-point scale at all study visits except visit 1. Each line represents an individual dog (*n* = 13). Body condition scores are integers, but some lines have been offset slightly to allow individuals to be distinguished

Change in bodyweight over time for individual dogs is shown in Fig. 3a. Between baseline and visit 3 (*n* = 11), body weight was unchanged in one dog, increased in a further eight dogs by between 0.10 kg and 4.0 kg (2.3-15.4% gain), and decreased in the remaining two dogs by 0.10 kg (9.8 kg to 9.7 kg, 1.0% loss) and 0.50 kg (6.8 kg to 6.3 kg, 7.4% loss), respectively. Of the two dogs that withdrew from the study before visit 3, body weight had increased by 1.8 kg (27.0 kg to 28.8 kg, 6.7% gain) in one and decreased by 0.2 kg (27.2 kg to 27.0 kg, 0.7% loss) in the other. There was no effect of visit on bodyweight (*P* = 0.338).

Body condition score was evaluated at visits 0, 2 and 3, and the median (range) was 3 (2–4), 4 (2–4) and 4 (3–5) at those visits, respectively. Change over time in BCS for individual dogs is shown in Fig. 3b. Body condition score was stable or improved in all dogs except one, which had lost 7.4% bodyweight associated with decrease in BCS from 4 to 3. However, the CIBDAI score of this dog decreased from 15 (extremely severe CE) at visit 0, to 3 (insignificant disease) at visit 3. Overall, a significant effect of visit on BCS was evident (*P* = 0.002), with pairwise comparisons showing BCS to be greater at visit 3 compared with visit 0 (*P* = 0.001) and at visit 3 compared with visit 2 (*P* = 0.041).

Appetite was reported to be abnormal in nine dogs, at visit 0, prior to commencing the study diet. At the time of the last report during study participation, appetite was described as normal in eight of these dogs, and abnormal in the ninth dog. Of the two dogs with normal appetite at visit 0, one had a normal appetite at the last study visit and the other had a reduced appetite unless the dry food was softened. There were no baseline records of appetite for the remaining two dogs; at the last study visit, one had a normal appetite, the other an abnormal appetite. Details for appetite at all study visits are provided in Additional file 1.

### Clinicopathological investigations

On haematological and clinical chemistry analyses, occasional results were outside the laboratory reference intervals (Table 4). The only exception was serum albumin concentration, which was less than reference interval (Biovet 23–39 g/L; Vebio 30–40 g/L) in 10/13, 9/12 (biochemical data were not available for one dog at this visit), and 6/11 dogs at the screening visit, visit 2 and visit 3, respectively; the median albumin concentration at these visits was 28 g/L (26–35), 29 g/L (22–32) and 29 g/L (25–37), respectively. At the screening visit, median serum folate concentration was 7.0 ng/mL (4.0 to > 24.0) and was less or greater than the laboratory reference interval (4.8–13 µg/L or 7–39 nmol/L depending upon the laboratory) in 3/13 and 4/13 of dogs tested, respectively. Serum cobalamin concentration at screening was greater than the reference interval (250–900 ng/mL or 150–700 pmol/L depending on the laboratory) in 1/13 dogs, and was less than the reference interval in 5/13 dogs, three of which were supplemented with subcutaneous cyanocobalamin (Vitamin B12 Delagrange 1000 µg/ml) at a dose of 50 µg/kg once weekly for 6 weeks. In a further two dogs, serum cobalamin concentrations were within reference interval but < 400 ng/L (recently suggested to be suboptimal in dogs with CE [36]).

**Table 4:** Haematological variables and biochemical analytes outside of the laboratory reference interval in dogs with confirmed chronic enteropathy

Variable	Screening visit (*n* = 13)		Visit 2 (*n* = 12) ^a^		Visit 3 (*n* = 11)	
	Above	Below	Above	Below	Above	Below
Erythrocyte count	1	0	1	0	1	0
Haemoglobin	1	0	0	1	2	1
Haematocrit	5	0	4	0	4	0
MCHC	1	0	1	0	0	0
MCH	2	1	1	2	0	3
MCV	7	0	5	0	7	0
White blood cell count ^b^	0	2	2	1	0	1
Neutrophil count ^b^	0	1	1	1	0	1
Eosinophil count ^b^	0	1	1	2	0	1
Basophil count ^b^	0	0	1	0	0	0
Lymphocyte count ^b^	0	0	2	0	0	0
Monocyte count ^b^	0	1	1	0	0	0
Platelet count	0	3	0	1	0	0
Urea	2	1	0	1	0	0
Creatinine	0	0	0	0	0	1
Gamma-glutamyl transferase	0	0	1	0	1	0
Aspartate aminotransferase	1	0	1	0	1	2
Alanine aminotransferase	2	0	0	0	1	0
Alkaline phosphatase	3	0	2	0	1	0
Glucose ^c^	3	0	4	0	4	1
Total protein	0	9	0	8	0	2
Albumin	0	10	0	9	0	6
Total bilirubin	0	0	1	0	0	0
Calcium	0	3	0	2	0	3
Phosphate	0	1	2	3	2	0
Cholesterol	1	1	2	0	3	0

All results represent the number of dogs with a result either above or below the reference interval of the respective laboratory. For full details, please see Additional File 1. Please note that blood samples were not taken at visit 1. ^a^ One of the 13 dogs in the study did not have haematology or biochemistry data at this timepoint (visit 2), as a result of being withdrawn due to administration of prednisolone for a neurological condition. ^b^ White blood cell counts were not available in one dog at visit 3 due to degeneration of the samples. ^c^ It was not possible to determine the glucose concentration for one and two dogs at visits 0 and 3, respectively, because the blood samples were not appropriately handled. MCHC: Mean corpuscular haemoglobin concentration; MCH: Mean corpuscular haemoglobin; MCV: Mean corpuscular volume

### Faecal score

Median faecal scores were 1 (1–3), 4 (2–5), 4 (3–5) and 4 (3–5), at visits 0 (*n* = 13), 1 (*n* = 13), 2 (*n* = 13) and 3 (*n* = 11), respectively. A visit effect was evident in these dogs (*P* < 0.001), and post-hoc analysis showed that faecal scores at visits 1, 2 and 3 were each greater than at visit 0 (*P* < 0.001 for each comparison). By the end of the study, faecal consistency was ‘normal’ in 6 dogs, ‘soft but formed’ in 3 dogs, and ‘hard and dry’ in 2 dogs (Table 5).

**Table 5:** Faecal scores over time in dogs with confirmed chronic enteropathy

Score	Description	Visit 0 (*n* = 13)	Visit 1^a^ (*n* = 13)	Visit 2^a^ (*n* = 13)	Visit 3^a^ (*n* = 11)
1	Liquid diarrhoea	7 (54)	0 (0)	0 (0)	0 (0)
2	Mostly unformed loose stools	5 (38)	1 (8)	0 (0)	0 (0)
3	Formed but soft stools	1 (8)	2 (15)	2 (15)	1 (9)
4	Formed, easy to pick up, optimal faeces	0 (0)	8 (62)	9 (69)	8 (73)
5	Formed, dry and hard faeces	0 (0)	2 (15)	2 (15)	2 (18)

Faeces was scored by owners using a semi-quantitative, 5-category faecal scoring system based on visual characteristics [57]. Overall there was a significant effect of visit on faecal score (*P* < 0.001). ^a^Faecal scores at visits 1, 2 and 3 were each higher than at visit 0 (*P* < 0.001 for each comparison)

## Discussion

In the current study, a second-generation hydrolysed diet containing poultry-feather-derived amino acids and oligopeptides was used in the successful management of 13 dogs with CE. The eligibility criteria ensured that dogs with CE were only recruited if they had not responded to previous dietary trials (including low residue, single-source protein and hydrolysed options). In addition, all of the confirmed CE cases had failed to respond to antibacterial therapy. This lack of response to previous therapies, along with the duration (median 10.5 months) of clinical signs was consistent with CE [16] which, according to the CIBDAI (median 9), was of a moderate-to-severe nature. Our results indicate the potential value of this amino acid- and oligopeptide-based diet in dogs with CE and no moderate or marked PLE that might otherwise be considered for immunosuppressant therapy on account of the failure of previous diet and antibacterial trials. Given that they were classified as having clinically insignificant disease after 10 weeks on the study diet, these 11 dogs could be given a formal diagnosis of FRE.

Currently, the most common dietary strategies for the management of CE in dogs are elimination diets, usually single-source protein diets or diets based on hydrolysed proteins. Single-source protein diets are not inherently hypoallergenic; instead, they are formulated with one protein and one carbohydrate type, ideally ones the dog has not previously been fed. First-generation hydrolysed proteins undergo a moderate degree of hydrolysis, and diets based on these can be effective in managing canine CE [18, 29, 30]. The second-generation hydrolysate used in the current diet had undergone more extensive hydrolysis resulting in free amino acids (88%) and oligopeptides of < 1 Kd, i.e. 6–8 amino acids in length [33], with purified amino acids added to ensure a balanced amino acid composition. This oligopeptide and amino acid mix has been shown to be hypoallergenic in dogs [34], and does not provoke reactions in dogs known to be sensitised to chicken protein [35]. Thus, the formulation of the study diet is like that of purified diets used in nutritional research, which are highly digestible and bioavailable, thereby limiting the amount of undigested nutrients available for the gut microbiota [29, 37].

More than 10 diet studies in dogs with CE have been published over the past 10 years [2, 7], but most have not assessed outcomes of dietary change without concurrent antibacterials or immunosuppressive drugs, and not all studies initiated a sequential treatment trial. Overall, there is good evidence of the effectiveness of soy-based hydrolysed diets in canine CE. These have improved clinical signs, decreased clinical activity and led to weight gain [8, 29, 30, 38, 39], as well as modulating the microbiome [8] and improving ultrastructural duodenal lesions [39]. Such clinical benefits have been observed from as early as 2 weeks after diet initiation [8, 30] and response has been maintained for up to 3 years [29]. However, often the histories of CE interventions have not been well documented and, therefore, the exact nature of the CE in these cases, whether food-responsive, immunosuppressant-responsive or the result of something else, is not clear. Arguably, this is a limitation of the current study because there was no attempt to characterise the CE-associated food antigens, and we did not confirm the response by subsequent rechallenge with the original diet. As a result, it is unclear whether these cases of CE were caused by hypersensitivity to a food allergen or were the result of an alternative mechanism such as a food intolerance. Dogs might also have responded to the study diet for other reasons such as improved digestibility of the diet. In human studies a hydrolysed protein diet has been beneficial as an alternative to corticosteroid treatment in active Crohn’s disease patients [31], or has reduced the occurrence of symptoms in babies at risk of cow’s milk allergy [40].

Besides the clinical improvement seen, the diet proved to be palatable in all dogs, and no adverse effects of the diet were noted throughout the study. Furthermore, all dogs except one either maintained or improved their body condition. In the dog whose BCS did not improve, disease was classified as severe at baseline (CIBDAI score 15), with 10 episodes of diarrhoea daily, and there was a concurrent flea infestation, leading to anxiety and pruritus. As a result, this dog was reluctant to consume all its food, although this improved over time, in combination with an improvement in clinical signs (visit 3 CIBDAI score 3, clinically insignificant disease). Clinical signs also improved in the dogs whose weight and body condition improved. Most dogs that completed stage 2 responded satisfactorily to therapy (*n* = 10/11), defined as a decrease in CIBDAI score of ≥ 75%, a definition that has been used in previous trials in dogs with CE [41, 42]. The dog that did not achieve a 75% score reduction suffered from moderate clinical signs at the outset (CIBDAI score 8) and improved to the point of being clinically insignificant by visit 3 (CIBDAI score of 3), a decrease of 63%. One of the two dogs that were withdrawn at week 5 also showed a good response. This dog did not complete stage 2 because it required prednisolone treatment for an unrelated hindlimb neurological condition. By the time of withdrawal, faecal consistency had normalised and signs had improved from severe (CIBDAI score 11) to clinically insignificant disease (CIBDAI score 2).

Folate and cobalamin are measured in canine CE because hypocobalaminaemia is a common finding of prognostic significance [36, 43, 44], whilst either increased or decreased folate concentrations can arise from malabsorption or concurrent dysbiosis [43]. Five of the dogs in the current study had hypocobalaminaemia and, consequently, three received parenteral supplementation. It is possible that these dogs were responding to this therapy rather than due to the dietary management. Interpretation of results is further limited by the fact that other drugs were used, although except for diosmectite, this was sporadic. The fact that diosmectite was permitted is a possible study limitation, because it is known to have some anti-inflammatory properties, for example, attenuating the severity of dextran sulphate colitis in mice [45], and reducing serum inflammatory markers in a mouse model of Crohn’s disease [46]. In dogs, diosmectite decreases the time to resolution of chemotherapy-induced episodes of diarrhoea [47]. However, to the authors’ knowledge, there are no reported studies showing a benefit of diosmectite in the management of CE in dogs. Of the four dogs that received diosmectite in this study, three had a history of previous treatment and one was treated with a single dose only. There was no clear difference in response between dogs treated with diosmectite during the study and those that were not. Taken together, these results suggest that diosmectite was not the main reason for the clinical response observed.

Besides those discussed above, the study has several other limitations that should be considered. First, the number of cases included was small and a sample size calculation was not performed, although the significant clinical responses suggest that the study was sufficiently powered for the outcomes studied. Second, this was an open-label study and there was no control group (e.g., comparison with other diet type). Therefore, these preliminary findings should be confirmed by a prospective and randomised double-blind study in a larger group of dogs suffering from CE.

Third, the criteria used for PLE (albumin < 20 g/L, total protein < 55 g/L) were similar to those used in two previous studies [32, 48], and were decided by agreement amongst study investigators to ensure standardisation amongst centres. However, given the range in albumin and total protein concentrations reported previously for PLE [49], it might have meant that some mild cases of PLE were still included. Conversely, our decision to exclude more severe PLE cases might have created a possible selection bias for less severe disease. That said, the cases were otherwise representative of dogs with CE referred to specialist gastrointestinal clinics, and all dogs had failed to respond to conventional therapies, although a range of previous diets were used, and these differed by dog.

Fourth, we made no attempt at a food challenge to confirm the role of diet in the development of CE; such confirmation would have been helpful but, as is commonly the case in clinical practice, none of the owners agreed to a provocation test after achievement of clinical remission (CIBDAI score reduction ≥ 75% in 10/11 dogs, CIBDAI ≤ 3 in 11/11 dogs). Fifth, resting cortisol was not measured in all cases, meaning that atypical hypoadrenocorticism might have been missed. However, this condition is known to be uncommon in dogs, comprising only 4% of dogs presenting with chronic gastrointestinal disease [50]. Sixth, there was no endoscopic re-evaluation or long-term follow-up further limiting our ability to confirm the nature and extent of the response; the 10-week study design was drafted when elimination diets for gastrointestinal disorders were generally trialled for a shorter duration than the current recommendation of at least 8 weeks [51]. Seventh, in some dogs, it was not possible to collect biopsies from all intestinal regions (e.g., duodenum [1 dog]; ileum [5 dogs]; colon [4 dogs]) and, as a result, it is possible that one or more of these dogs might have had small-cell lymphoma. In dogs, this condition can be low-grade in severity, and prolonged survival times can be seen, although treatment typically involves a combination of steroids and alkylating agents [52], or either chemotherapy or surgery [53]. The fact that the dogs with the occasional missing biopsies all improved rapidly following the dietary change, and their CIBDAI score decreased, makes lymphoma less likely but not impossible. Finally, the lack of long-term follow-up also meant that we were unable to test whether dogs would revert to their previous diets after complete clinical remission.

## Conclusions

In this open-label, uncontrolled clinical trial, dogs with CE that had previously failed to respond to both dietary management and antibacterials, had a good clinical response when fed an expanded dry diet containing oligopeptides and amino acids as the only protein source. These preliminary findings confirm the important role of diet in dogs with CE but require confirmation with a larger prospective study.

## Materials and methods

### Study design and outcome measures

This study was a 10-week, prospective, multicentre, unmasked and uncontrolled (i.e., single group), 2-stage field trial to determine the efficacy of an extensively hydrolysed protein diet in reducing the clinical signs of CE in dogs. A research protocol was prepared before the study commenced, but this protocol was not pre-registered. The individual dog was the experimental unit within the trial. The primary outcome measure was clinical response, based on changes in CIBDAI, which correlates with clinical activity of CE in dogs [54], and successful treatment was defined as a reduction in CIBDAI score of at least 75% [41, 42]. Secondary outcome measures included changes in faecal consistency, body weight, BCS and appetite. Results are presented only for dogs that had confirmed CE and participated in both study stages, as detailed in Fig. 1 and explained in the sections on visits and eligibility criteria below.

### Study sites and dates of study

There were four study sites in France (École Nationale Vétérinaire d’Alfort, Alfort; National Veterinary School of Toulouse, Toulouse; Clinique Alliance, Bordeaux; and Clinique Aquivet, Eysines) and one in Canada (Centre Vétérinaire Daubigny, Quebec City, Quebec, Canada). Dogs were screened and enrolled between 4 February 2013 and 21 February 2014, and study follow-up occurred between 19 February 2013 and 15 May 2014. A sample size calculation was not performed. Instead, investigators attempted to recruit as many eligible cases as possible within the timeframe in order to achieve a study population size broadly similar to those in related studies [16, 18, 30].

### Ethical considerations

The study was approved by the Ethics Review Committee of Royal Canin SAS (protocol code RCIBD-2012, Nov 5, 2012). All owners were informed of the nature of the study and gave written consent for their dog to participate.

### Eligibility criteria

Stage 1 of the study included client-owned dogs presenting with signs of gastrointestinal disease of suspected small intestinal origin suggestive of CE (e.g., vomiting and diarrhoea), of at least 4 weeks’ duration. Endoscopic biopsies were obtained at visit 0 to determine if dogs were also eligible for stage 2 of the study. Only dogs that completed stage 1 and that had a definitive diagnosis of chronic enteropathy with histological evidence of small intestinal inflammation were eligible to continue to stage 2.

Eligibility criteria applicable to all dogs included a requirement for owners to give their informed consent for study participation as detailed above, and the availability of complete records covering the period of participation in the trial. Dogs needed to be healthy based on a general physical examination, a comprehensive medical history, clinicopathological investigations and abdominal ultrasonography (see below). This meant that dogs with systemic disease (e.g., anaemia, chronic kidney disease, chronic hepatitis, neoplasia, hypoadrenocorticism), intestinal parasitic infections (based on faecal parasitology) and diagnosis of a specific gastrointestinal disease (e.g., moderate to marked protein-losing enteropathy [total protein concentration < 55 g/L, serum albumin concentration < 20 g/L or both]), pancreatitis [assessed by serum canine pancreatic lipase immunoreactivity and ultrasonography] and exocrine pancreatic insufficiency [based on low serum trypsin-like immunoreactivity]) were not eligible. Dogs were also not eligible if clinical signs were suspected to be solely of colonic origin, if there was evidence of a gastrointestinal foreign body or an intussusception (based on abdominal ultrasonography), or if the dog had had previous gastrointestinal surgery (including enterectomy and biopsy by laparoscopy or coeliotomy).

With respect to previous therapy, failure to respond to at least one dietary trial and one antibacterial trial (each of ≥ 14 days’ duration) was an inclusion criterion. Previous corticosteroid therapy was allowed, but this was not permitted in the 3 weeks prior to initial screening, or 30 days in the case of long-acting corticosteroid injections such as intra-muscular methylprednisolone. Finally, dogs were not eligible if they were unable to discontinue any flavoured medications or dietary supplements they were receiving.

### Visits

Initial screening comprised the assessments to determine eligibility for study stage 1, according to the criteria detailed above. Dogs were also treated empirically with 5 days of fenbendazole at 50 mg/kg every 24 h *per os* (Panacur^®^, Intervet, France and Canada), before stage 1 commenced.

Stage 1 comprised two visits: visit 0 occurred no more than 7 days after the initial screening, whilst visit 1 was conducted approximately two weeks (14 ± 2 days) after visit 0. At visit 0, gastrointestinal endoscopy was performed, including procurement of gastric and intestinal biopsies, and the study diet was commenced. At visit 1, the results of gastrointestinal histology were reviewed, and dogs that met Stage 2 eligibility criteria continued in the trial.

Stage 2 of the trial commenced immediately after visit 1 and comprised two further visits (visits 2 and 3), which were conducted approximately 5 weeks (35 ± 2 days) and 10 weeks (70 ± 2 days) after visit 0, respectively. Dogs entering stage 2 continued to receive the study diet for a further 8 weeks without interruption.

### Test diet and feeding protocol

The test diet was a dry, extruded food (Anallergenic™ Canine Formula, Royal Canin Veterinary Diets™, Aimargues, France; Table 6), formulated with poultry-feather protein (keratin) that had been extensively hydrolysed with hydrochloric acid to produce free amino acids (88%) and oligopeptides less than 1 kilodalton (6–8 amino acids) [33]. There were no other protein sources except for purified amino acids that were added to balance the essential amino acid composition.

**Table 6:** Composition of the study diet

Diet component	Amount ^a^
Metabolisable energy content ^b^	16,250 kJ / 3884 kcal
Moisture, g	16.7
Protein, g	46
Crude fat, g	43
Dietary fibre, g	15
Crude fibre, g	5.7
Crude ash, g	22.7
Nitrogen-free extract, g	124
Ingredients	Maize starch, poultry feather hydrolysate with low molecular weight (source of L- amino acids and oligopeptides), copra oil, soya oil, minerals, vegetable fibres, chicory pulp, fructo-oligosaccharides, fish oil, mono- and diglycerides of fatty acids esterified with citric acid, animal fat, marigold extract (source of lutein), vitamins

^a^ Besides metabolizable energy content, all amounts are expressed as grams per Mcal of metabolizable energy content of the diet. ^b^ Metabolizable energy content (in kJ / Kcal) per kilogram of food, as fed, calculated using the predictive equation recommended by the National Research Council [56]

Dogs were fed the study diet for a period of 10 weeks from visit 0 onwards. The daily ration that owners were asked to feed was calculated according to the manufacturer’s recommendations, and was based upon both the dog’s current body weight and BCS [55]. Accordingly, the amount fed ranged from 460 kJ per kg^0.75^ per day (110 Kcal per kg^0.75^ per day), for dogs that were at or above their ideal weight (BCS ≥ 4/9), to 523 kJ per kg^0.75^ per day (125 Kcal per kg^0.75^ per day), for dogs that were below their ideal weight (BCS < 4/9) [56]. Owners were instructed to ensure that no other food was eaten and to provide free access to water but no other liquids. Dogs were reweighed at each visit and, if dogs had lost ≥ 5% of their bodyweight, the food allowance was increased by 10%.

### Monitoring of clinical signs

A general physical examination was performed at the pre-study screening visit and at each of the four scheduled study visits (visits 0 to 3). Dogs were weighed at all study visits using the same electronic weigh scales at each site, and BCS was assessed with a 9-point scale [55] at all study visits except visit 1.

Owners completed a questionnaire (Additional file 2) every 2 weeks whilst their dog was on the study, where they recorded details of their dog’s gastrointestinal signs, appetite, coat condition and signs of pain. The questionnaire included a semi-quantitative, 5-category faecal scoring system based on visual characteristics (Additional file 2 [57]), and owners were asked to record the lowest score of faecal quality observed during the preceding 2 weeks. The apparent palatability of the study diet was assessed using a semi-quantitative, 5-category system (1: very poor [e.g., complete food refusal]; 2: poor [e.g., turns over the bowl ± eats the food over several meals]; 3: average [e.g., eats but easily disturbed or hesitates initially, and then eats); 4: good [eats but does not rush to the bowl]; 5: very good [rushes to the bowl and eats quickly]). Owners were also asked to record any alterations in faecal consistency, the frequency of defaecation, and the presence of other signs including vomiting, flatulence, tenesmus and melaena. Owners monitored the wellbeing of their dogs throughout the study and were instructed to alert the attending clinician to any concerns.

The attending clinician reviewed the clinical signs at each study visit and assigned a CIBDAI score based on these observations and the responses recorded in the owner questionnaire [58]. Full details of CIBDAI have been published previously [58]; briefly, six gastrointestinal signs (attitude and activity, appetite, vomiting, stool consistency, stool frequency and weight loss) were scored for severity (from 0 to 3). Scores were summed to give a composite evaluation of clinical signs: 0–3, not clinically relevant; 4–5, mild; 6–8, moderate; ≥9, severe.

### Adverse events and early trial discontinuation

Any suspected adverse events were recorded and, if thought to be the result of the study diet, participation was suspended immediately. Treatment with diosmectite and parenteral cobalamin were permitted during the study. Where response to therapy was deemed to be poor at follow-up visits, additional therapy could be added at the discretion of the attending clinician. However, dogs that were administered systemic corticosteroids or antibacterials had to be withdrawn from the study. Participation could also be stopped if an enrolled dog developed an unrelated condition. Owners could withdraw their dog from the study at any stage, without providing a reason, if they wanted to.

### Clinical pathology

Fasting blood samples were taken for haematological and clinical chemistry analyses at the initial screening as well as at visits 2 and 3. Haematology, clinical chemistry, urinalysis (including dipstick and specific gravity measured by refractometry), serum trypsin-like immunoreactivity (TLI), canine pancreas-specific lipase, folate and cobalamin and faecal parasitology (faecal flotation and smears) were performed at Vébio (Arcueil, France) for the Alfort, Bordeaux and Eysines sites, Laboratoire de parasitologie and Laboratoire de biologie medicale, École Nationale Vétérinaire de Toulouse (ENVT, Toulouse, France) for the Toulouse site, and Biovet, (St-Hyacinthe, Canada) for the Quebec site. Resting cortisol concentration was measured if clinically indicated based on the decisions of the attending clinicians. For example, resting cortisol would be measured in dogs presenting with anorexia and weight loss, or where there were indirect haematological signs of atypical hypoadrenocorticism (lymphocytosis, eosinophilia); however, it was not mandatory in young, otherwise well dogs in good body condition where gastrointestinal signs had been present since acquired by the owner; in such cases, it was assumed that atypical hypoadrenocorticism would be unlikely. An adrenocorticotropic hormone (ACTH) stimulation test was performed if cortisol concentration was ≤ 50 nmol/L (or ≤ 55 nmol/L for the Toulouse site).

### Ultrasonography

Before enrolment in the study, abdominal ultrasonography was performed in all dogs to identify conditions that might lead to study ineligibility (see above). The equipment used varied by study site (Alfort, Bordeaux and Eysines: MyLab™ 60, Esaote SpA, Genova, Italy; Toulouse: Loqiq™ 7, GE Healthcare, Chicago, USA; Toulouse: MyLab™ 50, Esaote SpA).

### Gastrointestinal endoscopy and histology

A combined gastroduodenoscopy and ileocolonoscopy procedure was performed at visit 0 to confirm a diagnosis of canine CE. Multiple mucosal biopsies (at least 3–4 per accessible segment) were obtained from the stomach (body and fundus), duodenum, distal ileum and colon [59]. Endoscopy was performed with an Olympus GIF-Q-180 video gastroscope (Rungis, France; or, Olympus, Quebec, Canada) with a working channel of 2.8 mm, and biopsies were taken using single-use biopsy forceps (EndoJaw™ FB-210 K, OLYMPUS, Rungis, France). Histological slides were prepared from biopsies by the Laboratoire d’Anatomie Pathologique Vétérinaire du Sud-Ouest (LAPVSO, Toulouse, France), Vébio (Arcueil, France) or the Laboratoire d’Histologie et Anatomie Pathologique (ENVT, Toulouse, France) for the French sites, and by Histovet Surgical Pathology (Guelph, ON, Canada) for the Canadian study site. All slides were reviewed by two French-certified pathologists (Dr Frédérique Degorce-Rubiales and Dr Melanie Fine) using the scoring system developed by the World Small Animal Veterinary Association (WSAVA) Gastrointestinal Standardization Group [60].

### Data handling and statistical analysis

Data were entered into an electronic spreadsheet (Excel 97-2003 Workbook, Microsoft) and checked for errors before importing into an online open-access statistical language and environment (R, version 4.0.1 [61]) for analysis. Additional packages used for data analysis included ‘tidyverse’ (version 1.3.1 [62]), and ‘Lme4’ (version 1.1–29 [63]). The complete study data are presented in Additional file 1. Outcomes (CIBDAI, BCS, faecal score and palatability score) are summarised by median (range), and CIBDAI outcomes are also summarised as numbers of dogs within each severity category and number achieving treatment success. Faecal scores are also presented categorically. Clinicopathological variables (haematology and clinical chemistry) were evaluated according to whether the values were above, below or within the reference intervals specific to each laboratory. For dogs that participated in both study stages, one-way repeated measures ANOVA was used to evaluate the effect of visit on CIBDAI score, body weight, BCS and faecal consistency score. Data were log- or rank-transformed when appropriate to meet statistical model assumptions (e.g., normally-distributed residuals and homoscedasticity, based on graphical inspection of data distribution, Shapiro-Wilk test, skewness and Kurtosis; Additional file 1). When there was a significant effect of visit on an outcome, Tukey post-hoc tests were performed on pairwise comparisons between visits. Given the multiple comparisons, Tukey HSD was used to correct *P*-values for alpha risk inflation. The level of statistical significance was set at *P* < 0.05, and all comparisons were two-sided.

## Electronic supplementary material

Below is the link to the electronic supplementary material.

Supplementary Material 1: Additional file 1. Complete study data. This file contains complete clinical information from the dogs in the study. Data are organised into separate sheets of eligibility criteria, visit dates, IBD disease (endoscopy and histology), owner questionnaire information, haematology, biochemistry, laboratory reference intervals, protocol deviations (including diet and other therapies), previous dietary trials and summary of statistical analyses

Supplementary Material 2: Additional file 2. Questionnaire for pet owners. Complete questionnaire used to gather information from clients at each visit

## Data Availability

A file containing all anonymised research data supporting this article can be found in the additional information provided (Additional file 2).
