# Supplementary material for: An extensively hydrolysed protein-based extruded diet in the treatment of dogs with chronic enteropathy and at least one previous diet-trial failure: a pilot uncontrolled open-label study
Source: BMC Vet Res. 2025 Feb 15;21:68. doi: 10.1186/s12917-025-04528-y (PMC11830179; doi:10.1186/s12917-025-04528-y)
Supplement: Supplementary file 2 — Supplementary Material 2: Additional file 2. Questionnaire for pet owners. Complete questionnaire used to gather information from clients at each visit [file 12917_2025_4528_MOESM2_ESM.pdf]

Supplement 1. Questionnaire for pet owners

1. General behaviour and pain:

Does your animal show the following behaviours? Yes or no

a. Behaviour as usual – no change
b. Eating grass, licking the ground (pica)
c. Signs of acute pain
d. Prayer (antalgic) position (explained by the clinician at inclusion)
e. Change in its interaction with you

2. Coat condition

a. As usual
b. Worsened (less soft, less shiny, dandruff)
c. Improved
d. Other observations

3. Faecal quality

a. According to a scale of 1 (liquid diarrhoea) to 5 (hard and dry faeces), with 4 being optimal (see figure below): how would you best describe the faeces of your dog?
b. Prescence of fresh blood: Yes or no
c. Prescence of dark-black faeces: Yes or no

4. Other gastrointestinal signs

a. Tenesmus: Yes or no
b. Flatulence: Yes or n and frequency/day
c. Vomiting: Yes or n and frequency/day or week

5. Palatability of the diet

1. Very poor, dog refuses to eat the diet
2. Poor, turns around the bowl and eats the food in several meals
3. Average, easily disturbed, hesitates, but eats

4. Good, eats but does not rush to the bowl

5. Very good, rushes to the bowl and eats quickly

Figure. Faecal quality scale

Directions for use. Score the stools of each dog from 1 (liquid) to 5 (formed and dry). When the consistency of the stools is not homogeneous, record the lowest score.

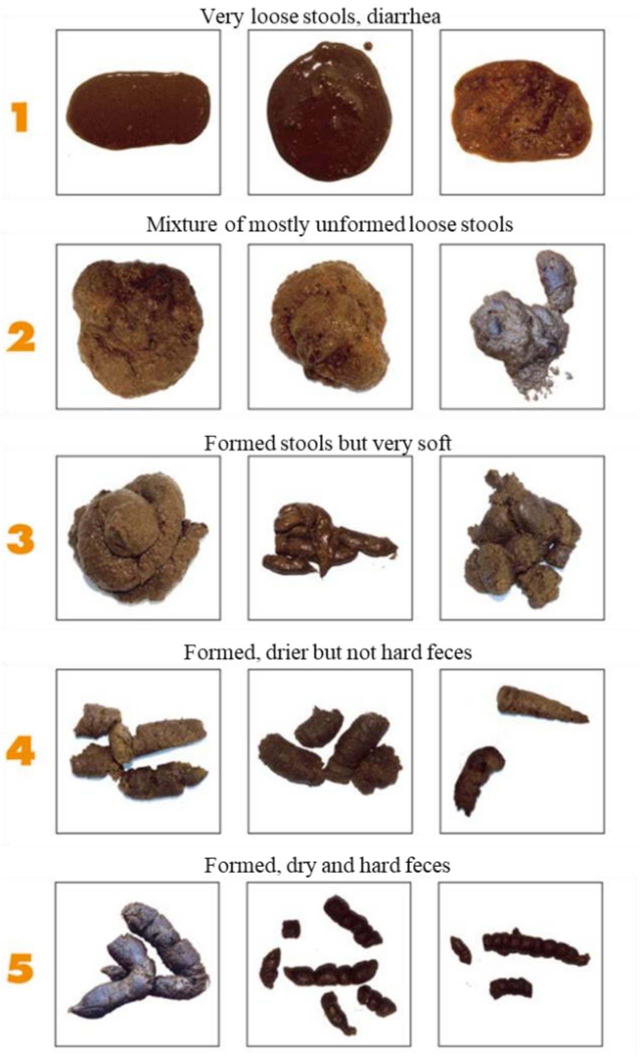

(1 – liquid diarrhoea; 2 – mostly unformed loose stools; 3 – formed but soft stools; 4 – formed, easy to pick up optimal faeces; and 5 – formed, dry and hard faeces) (Supplementary figure 1). When the consistency of the stools was variable, the lowest score was recorded.
